# Dynamic probe of ZnTe(110) surface by scanning tunneling microscopy

**DOI:** 10.1088/1468-6996/16/1/015002

**Published:** 2015-01-13

**Authors:** Ken Kanazawa, Shoji Yoshida, Hidemi Shigekawa, Shinji Kuroda

**Affiliations:** Faculty of Pure and Applied Sciences, University of Tsukuba, Tsukuba 305-8573, Japan

**Keywords:** scanning tunneling microscopy, atom manipulation, ZnTe, semiconductor surface

## Abstract

The reconstructed surface structure of the II–VI semiconductor ZnTe (110), which is a promising material in the research field of semiconductor spintronics, was studied by scanning tunneling microscopy/spectroscopy (STM/STS). First, the surface states formed by reconstruction by the charge transfer of dangling bond electrons from cationic Zn to anionic Te atoms, which are similar to those of IV and III–V semiconductors, were confirmed in real space. Secondly, oscillation in tunneling current between binary states, which is considered to reflect a conformational change in the topmost Zn–Te structure between the reconstructed and bulk-like ideal structures, was directly observed by STM. Third, using the technique of charge injection, a surface atomic structure was successfully fabricated, suggesting the possibility of atomic-scale manipulation of this widely applicable surface of ZnTe.

## 1. Introduction

The characterization of semiconductor surfaces is important not only for fundamental scientific research but also for the application of nanotechnology. Group IV (Si and Ge) and III–V (GaAs, GaP and InSb) semiconductor surfaces have been intensively studied, particularly from the viewpoint of developing novel electronic devices. ZnTe, which we focus on in this study, is a II–VI semiconductor with a relatively wide direct band gap and is expected to be applied for optical devices such as green light-emitting diodes [1] and THz light generators and detectors [2, 3]. In addition, ZnTe is also commonly used as a substrate for CdTe self-assembled quantum dots [4]. Furthermore, it is used as a host material of diluted magnetic semiconductors (DMS), which are promising materials in the research field of semiconductor spintronics [5, 6]. For example, (Zn,Cr)Te is well known for its intrinsic room-temperature ferromagnetism, which is realized when the Cr composition is higher than 20% (Zn_0.8_Cr_0.2_Te).

**Figure 1.**
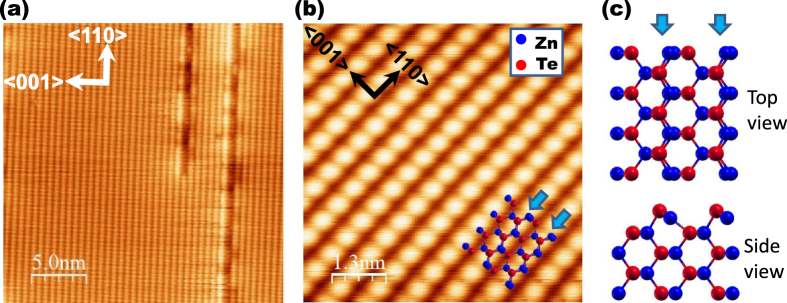
(a) STM image of ZnTe(110) surface (*V*_s_ = −1.5 V, *I*_t_ = 60 pA). (b) High-resolution image of ZnTe(110) surface (*V*_s_ = −2.0 V, *I*_t_ = 20 pA). (c) Schematic structures of ZnTe(110) surface.

Comparing to group IV or III–V semiconductors, however, the ZnTe surface has not been well comprehended. Moreover, reported studies are mainly concerned with static surface properties such as atomic structure and electronic ground state, but dynamic phenomena on the surface have been largely uncertain. ZnTe(110) surface states, which are the topic of this paper, have been studied by low-energy electron diffraction [7], scanning tunneling microscopy (STM) [8, 9], angle-resolved photoelectron spectroscopy (ARPES) [10], electron energy loss spectroscopy (EELS) [11] and density functional theory (DFT) calculations [12]. These results have revealed that the topmost cationic and anionic atoms are slightly displaced toward the inside of the crystal and vacuum from the ideal (110) structures of truncated bulk crystal, respectively, and the atomic arrangement of ZnTe(110) surface is fundamentally similar to that of III–V semiconductor surfaces (hereafter referred to as the reconstructed surface). The STM observations, however, showed that this surface is unstable and friable, i.e., line defects tend to be generated along the 〈110〉 direction (figure 1(a)) when a clean surface is obtained by cleavage [8, 9]. In addition, when the surface is scanned by STM with a positive sample bias voltage, surface atoms are often desorbed from the surface [8]. These characteristics are unique and markedly different from those of IV and III–V semiconductor surfaces.

With the reduction of device size, the development of composite materials with multiple functions is required. Control of the surface atomic and electronic structures, particularly that of atomic-scale defects, has become a key technology to meet the requirements. Therefore, to exploit the characteristics of ZnTe, it is important to clarify the structures and the associated phenomena of its surface. For this purpose, we have carried out scanning tunneling microscopy and spectroscopy (STM/STS) analysis of this surface. In addition to confirming the charge transfer on this surface, oscillation in tunneling current, which is considered to reflect the positional fluctuation of the topmost Zn atom induced by charge injection was observed. Furthermore, by applying this mechanism, we have succeeded in fabricating atomic structures on the surface. Such fabrication is expected to play a role, for example, in modulating the local potential of a surface to design functional devices with various electronic properties.

## 2. Experimental methods

A p-type ZnTe single crystal doped with phosphorus of a concentration of 10^18^ cm^−3^ was used as a sample in this study. All STM/STS measurements were performed with an Omicron low-temperature scanning tunneling microscope under ultrahigh-vacuum conditions (<10^−8^ Pa) at 8 K and 77 K using an electrochemically sharpened W tip (*ϕ* = 0.3 mm). A ZnTe(110) surface was obtained by cleaving the sample in a high vacuum (∼1 × 10^−5^ Pa).

## 3. Results and discussion

Figure 1(a) shows a typical STM image obtained at a sample bias voltage of *V*_s_ = −1.5 V. Since occupied lone pair states are formed on the topmost Te atoms due to charge transfer from topmost Zn atoms, the Te atoms can be imaged as bright points at a negative sample bias voltage. In addition to the bright lines of Te atoms along the 〈110〉 direction, several dark lines along the same direction were observed. According to the previous study [8], these dark lines are line defects caused by the absence of topmost atoms and are intrinsic defects of the ZnTe(110) surface, as mentioned above. From the different brightness on the both sides of the dark-line defects, we can determine the indexes of the in-plane crystallographic axes on the surface (the distinction between [110] and [−110] axes), as shown in figure 1(a) [9]. And we could determine the correspondence of the STM image to the atomic arrangement on the surface (figure 1(c)) as shown in figure 1(b).

**Figure 2.**
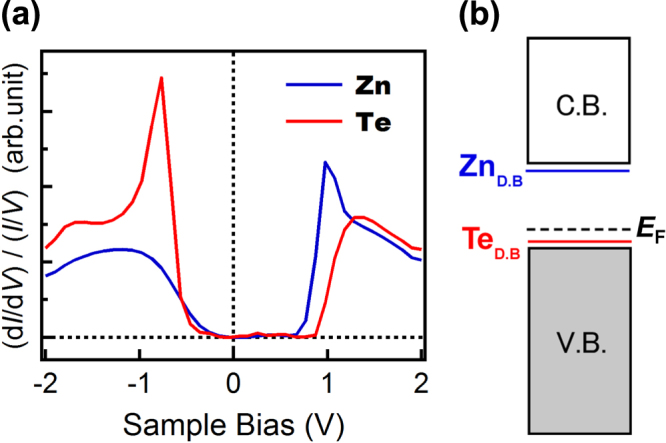
(a) Normalized differential conductance (NDC)—sample bias voltage spectra obtained above a Zn atom (blue) and a Te atom (red). The setpoint voltage and current were *V*_s_ = −2.0 V and *I*_t_ = 20 pA, respectively. The peaks shown in the blue (+1.0 eV) and red (−0.8 eV) lines indicate the energy levels of the dangling bond states of the surface Zn and Te atoms, respectively. (b) Schematic of the ZnTe band structure derived from the NDC-*V*_s_ spectra.

To investigate the surface electronic structure in more detail, we carried out STS measurement. Figure 2 shows the results of normalized differential conductance (NDC) analysis, which reflects the local density of states (LDOS). NDC-*V*_s_ curves were measured on topmost Zn (blue) and Te (red) atoms, respectively. There are differences between the two curves, particularly at the rising edges from the semiconductive gap region near *V*_s_ = 0. At the rising edge at a positive (negative) sample bias voltage, the NDC-*V*_s_ spectrum obtained at the Zn (Te) position exhibits a higher peak than that obtained at the Te (Zn) position. These results can be comprehensively explained by the mechanism that the empty and occupied dangling bond states are formed through the charge transfer from cationic Zn to anionic Te associated with the surface reconstruction. Similar charge transfer is well known to occur on several semiconductor surfaces such as Si(100) dimer and GaAs(110) reconstructed surfaces, which has been confirmed by STM observation with atomic resolution [13, 14]. The STS analysis has confirmed the surface states of ZnTe(110) in the real space. The observed energy levels correspond to the surface states studied by ARPES [10], EELS [11], and DFT [12], which have suggested that the anionic Te (cationic Zn) dangling bond state is located near the valence band maximum (conduction band minimum), as shown in figure 2(b). Here, the energy gap of the host ZnTe was determined from the distance between the Zn (−1.0 V) and Te (+1.3 V) peaks in the spectra, because we have to remove the influence of their surface states. The gap energy obtained is about 2.3 eV, which is close to the previously reported energy gap of ZnTe (2.39 eV at 4.2 K).

In this STS measurement, a small current set point (−2 V, 20 pA), which was used to observe the STM image in figure 1(b), was chosen to reduce the effect of the surface instability reported in an STM measurement for the positive sample bias voltage region. In fact, no remarkable phenomenon related to the instability was observed during the STS measurements. However, with a larger tunneling current setpoint, the surface instability appeared. Therefore, next we investigated the instability observed during the STM measurement at a positive sample bias voltage in more detail. To observe the expected dynamics on the surface, we studied the effect of the electron-injection from the STM tip to the sample at 8 K with the tip position fixed at meshed points during the two-dimensional scan over the surface.

**Figure 3.**
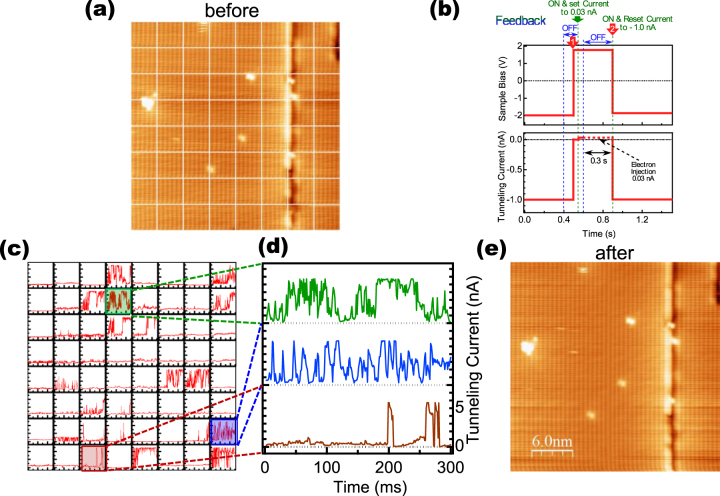
(a) STM image obtained at 8 K (*V*_s_ = −3.0 V, *I*_t_ = 1.0 nA) with 8 × 8 mesh in which time-dependent STM measurements were carried out. (b) Sequence used in measurement of temporal change of the tunneling current (*t*_i_: electron injection time). At first we opened the STM feedback loop and the sample bias voltage was changed to a positive value of *V*_s_ = +1.5 V (red arrow 1). Then, we closed the feedback loop at the set point with a tunneling current of +0.03 nA (green arrow) to start injection of electrons from the tip to the sample (injection). Almost at the same time, we opened the feedback loop again to fix the tip and started measurement of tunneling current. After an electron injection, the bias voltage was returned to *V*_s_ = −2.0 V (red arrow 2). (c) Measurement of temporal change of the tunneling current obtained for the 8 × 8 mesh shown in (a). (d) Three typical plots in a magnified scale selected from those in (c). The spectra shown in green, blue and red respectively correspond to those in the squares in (c) indicated by the same colors. (e) STM image obtained after time-resolved STM measurements shown in (c) (*V*_s_ = −3.0 V, *I*_t_ = 1.0 nA).

Figure 3(a) shows an STM image of the surface with an 8 × 8 mesh in each of which the STM electron-injection sequence shown in figure 3(b) was carried out as follows. (1) during an STM scan with the set-point conditions (sample bias and current) of *V*_s_ = −2.0 V and *I*_t_ = −1.0 nA, the tip was held at a certain line crossing in the mesh (*t* = 0). (2) After opening the STM feedback loop, the sample bias voltage was changed to a positive value of *V*_s_ = +1.5 V (red arrow 1), at which the tunneling current usually became very small reflecting the *I*–*V* character of this surface. (3) Then, the feedback loop was closed at the set point with a tunneling current of +0.03 nA (green arrow). In this step, the distance between the STM tip and the sample became small and electronic injection from the tip to the sample started to occur (injection). After a very short time, we open the feedback loop again to fix the distance between the tip and the surface and started measurement of tunneling current at the same time. (4) After an electron injection time of *t*_i_ = 0.3 s, the bias voltage was returned to *V*_s_ = −2.0 V (red arrow 2).

Figure 3(c) shows the tunneling current as a function of time measured in each 8 × 8 grid in figure 3(a). The origin, *t*_mes_ = 0, is the time when the tunneling current was set to +0.03 nA. Variations in the tunneling current can be seen. Figure 3(d) shows three typical examples of data selected from figure 3(c). The current appears to oscillate between the original ‘low-current’ state and another ‘high-current’ state. No damage was observed in the STM image obtained after sequential measurements, as shown in figure 3(e), which indicates that, during the sequence, the charge injection of this condition induced only current oscillation without any destructive contact or scratching of the surface with the STM tip.

As a possible reason of the current oscillation, we may consider first the simple reflection of a temporal occupying event of electrons at the surface such as the Coulomb-blockade, which, however, is not consistent with our experimental results. If Coulomb-blockade was formed, we should observe the effect of the ‘staying electron’ as the drop of the tunneling current. However, we observed the jump-up of tunneling current from the original ‘low-current’ state at the start of the injection of the tunneling electron.

Comparing the current oscillation with the unstable STM observation with a positive sample bias voltage as reported by the earlier study [8], a possible phenomenon to be able to explain the current oscillation is a structural variation between the original surface-reconstructed structure and another structure, which causes the ‘high-current’ state, i.e., the STM sequence induces a structural change of the topmost Zn or Te atom toward the vacuum side from the reconstructed structure.

What causes the structural change? A possible mechanism to excite the structural change is the effect of inelastic tunneling which is widely observed. However, any current oscillation was not observed in a similar sequence with a negative sample bias voltage, which should appear in the case of inelastic tunneling: inelastic tunneling occurs at the same positive and negative bias voltages.

**Figure 4.**
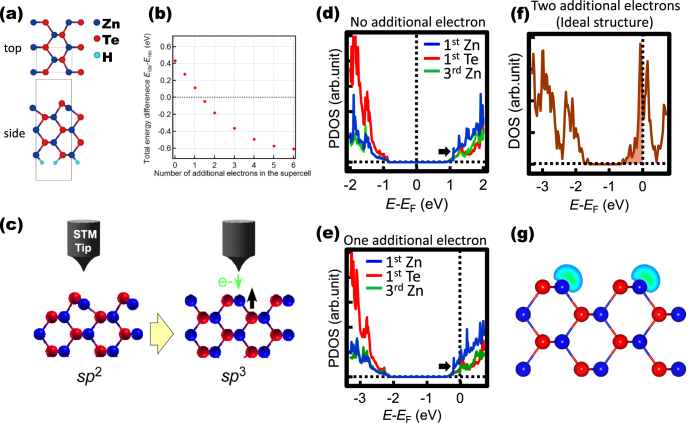
(a) Schematic illustration of the ZnTe structure used for the DFT calculation. (b) Total energy difference between the ideal and reconstructed surface structures (*E*_ide_−*E*_rec_) as a function of the number of injected electrons. (c) Schematic diagrams showing atom manipulation by charge injection. Electrons injected from the STM tip induce the displacement of the topmost Zn atoms toward the vacuum side. (d) and (e) Calculated partial density of states (PDOS) for the topmost Zn and Te atoms and the Zn atom located in the third (110) layer of the reconstructed surface with no (d) and one (d) additional electron. (f) DOS calculated for the ideal surface structure with two additional electrons. (g) Calculated spatial distributions of the LDOS integrated over *E*_F_−0.2 eV < *E* < *E*_F_ (*E*_F_: Fermi energy) for the ideal surface structure with two additional electrons.

In order to establish a possible interpretation for the surface dynamics induced by the electron injection of the STM sequence, we carried out a DFT calculation using the Perdew–Burke–Ernzerhof generalized gradient approximation with the ABINIT code and plane-wave-based norm-conserving pseudopotentials. A supercell including a slab model with five ZnTe(110) layers was used for the calculation. The dangling bonds of atoms in the bottom layer were passivated by hydrogen atoms, as shown in figure 4(a). The energy cutoff of the calculation was 60 Ry. As the sampling k-points, we used 8 × 8 × 2 grids within the first Brillouin zone. Here, we focused on a possibility of the atomic displacement of the topmost Zn atom, because the Zn atom is considered to be most easily desorbed from the surface, referring to the previous study [9], which reports that the defects on the cleaved ZnTe(110) surface mainly consist of the absences of Zn only or Zn–Te pairs and not Te only. As a possible computational model to describe the atomic displacement of the surface Zn atom toward the vacuum side from the reconstructed structure, we consider the ideal (110) surface structure consisting of a truncated bulk structure. The stability of the surface was analyzed by comparing the total energy difference between the ideal and reconstructed surface structures in the neutral state and in charged states with up to six additional electrons per supercell.

Figure 4(b) shows the summary of the results of our DFT calculation, showing the total energy difference between the ideal and reconstructed surface structures (*E*_ide_−*E*_rec_) as a function of the number of additional electrons per supercell. As shown in the figure, the reconstructed surface is more stable than the ideal surface in the neutral state (without additional electrons). This result validates the surface reconstruction of this surface with the buckling of the Zn–Te structure, similar to the cases of the Si dimer and Ga–As structure appearing upon their surface reconstruction [13, 14]. On the other hand, the surface stability changes with the charge injection; the ideal configuration becomes more stable when the number of additional electrons is larger than 1.5. Considering the result of our theoretical study with the result of our experiment, in which the variations in the tunneling current was observed, these results may indicate that the electrons injected from an STM tip induce modulation of the surface structure; the charge injection into the surface region may induce an atomic oscillation of the surface Zn atoms between the reconstructed structure and a structure with displacement of the Zn atom toward the vacuum side as schematically shown in figure 4(c).

Furthermore, in order to discuss the effect of the electrons injected from the STM tip in more detail theoretically, we calculated partial density of states (PDOS) for the topmost Zn and Te atoms and the Zn atom located in the third (110) layer, which should correspond to the state far from the topmost surface, of the reconstructed surface with no and one additional electron. The results of the PDOS calculations show that the PDOS of the topmost Zn atom is the largest near the conduction band minimum in the neutral condition (figure 4(d)) and the state is occupied by one additional electron with the shift of the Fermi energy (*E*_F_) (dotted lines), as indicated by a black arrow (figure 4(e)). In addition, the theoretical results of the DOS (figure 4(f)) and spatial distribution of the LDOS integrated over *E*_F_−0.2 eV < *E* < *E*_F_ (figure 4(g)) calculated for the ideal surface structure with two additional electrons, which may cause the inversion of the surface stability, clearly indicate that the additional electrons are preferentially located at the topmost Zn atom of the ideal structure. These results are consistent with those of our STS measurement, in which we observed the Zn_DB_ state near the conduction band minimum, suggesting that the injected electrons tend to be located at the dangling bond states of the topmost Zn. Upon the occupation of the empty Zn_DB_ states by the additional electrons, the bonding state of the Zn atom with neighboring Te atoms may change from the flat *sp*^2^-like conformation to the *sp*^3^-like tetrahedral coordination. The distance between the STM tip and the Zn atom decreases upon the conformational change from the reconstructed structure to the displaced structure with Zn atom moved toward the vacuum side, producing the high state observed in tunneling current. According to our theoretical study, the conformational change is considered to be a possible mechanism for producing the current oscillation observed during the STM electron-injection sequence.

If the STM tip and the surface Zn atom are closely located, the electrons are injected to the dangling bond with a high possibility, and, the displacement should occur frequently. In this case, the ‘high-current’ state is supposed to be observed continually. Actually, comparing the details of the current oscillations shown in figure 3(c), the tunneling current in the top spectrum of figure 3(d) tends to be high, for example, and in the bottom spectrum the oscillation occurs less often. This difference is considered to be due to the relative lateral positions of the STM tip to that of a Zn atom below the tip. Since the measurement in each mesh was started without checking the relative positions, the STM tip might be located only slightly above the Zn atom in some cases, but at a much greater distance from the Zn atom in other cases.

**Figure 5.**
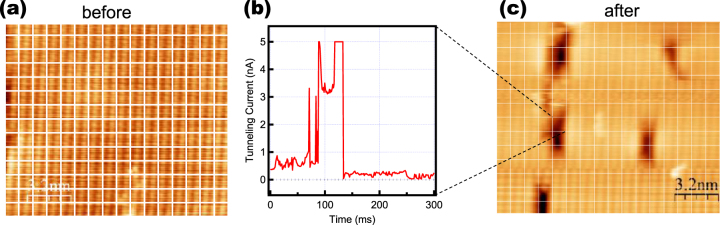
(a) STM image before the electron-injection sequence with *V*_s_ = +1.5 V and *I*_t_ = 0.3 nA (*V*_s_ = −3.0 V, *I*_t_ = 1.0 nA). (b) Temporal change of the current obtained during the electron-injection sequence carried out on the area indicated by dotted lines. (c) STM image of the surface defects generated by the electron-injection sequence (*V*_s_ = −3.0 V, *I*_t_ = 1.0 nA).

Next, we consider the relationship between the structural oscillations and the atomic desorption from the surface induced by STM observation at a positive sample bias voltage in the previous study. No damage reflecting the atomic desorption was observed with the measurement condition of the STS measurement (figure 1) and during the above STM sequence (figure 3) in this study. However, the atomic desorption occurred when we used the setpoint tunneling current of ten times larger, *I* = 0.3 nA. Figures 5(a) and (c) show the STM images obtained before and after the electron injection sequence shown in figure 3, which was carried out at the grid points of the mesh drawn in figure 5(a). Formation of defects is clearly shown in figure 5(c). When the surface Zn atom is displaced toward to the vacuum by the electron injection, tunneling current increases. In fact, much larger tunneling current, which exceeded the detection limits of 5 nA was observed during the electron injection as shown in figure 5(b), which might induce the atomic desorption as a result of an enhancement of the atomic oscillation.

Here, the appearances of the atomic deficiencies generated by the desorption process are quite similar to the intrinsic line defects on the cleaved ZnTe(110) surface recognized as a missing row of Zn atoms or Zn–Te pair, though the length of the generated defects are quite short. Therefore, it is reasonable to consider that the short defects arise from the position of the surface Zn atom, corresponding to the theoretical calculation, which supports the model that the charge injection from the STM tip induces the displacement of the surface Zn atom.

This result suggests that we should consider the experimental parameters to enhance the interaction between the STM tip and the surface atom during the electron injection, in order to induce the desorption of surface atoms. Specifically, possible reasons, which may influence the probability of the desorption, include the tunneling current, the distance, the electric field between the STM tip and the sample surface, and the local temperature.

To verify the mechanism, we carried out current injection under different conditions. Namely, we observed the surface and placed the STM tip immediately above a Zn atom, and then the sequence shown in figure 3(b) was carried out. The sample temperature was set at 77 K.

**Figure 6.**
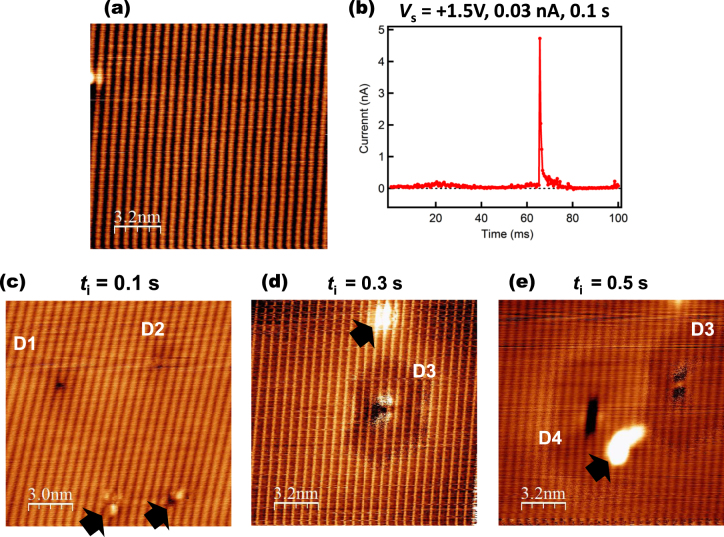
(a) STM image of a ZnTe surface obtained before the electron injection treatment (*V*_s_ = −3.0 V, *I*_t_ = 1.0 nA). (b) Typical temporal change of the current obtained during the treatment. (c), (d), (e) STM images after the treatment with three different electron injection times of *t*_i_ ((c) 0.1 s, (d) 0.3 s, (e) 0.5 s, *V*_s_ = +1.5 V, *I*_t_ = 30 pA). D1 to D4 are surface defects induced by each treatment. D3 in (d) and (e) indicate exactly the same defect. Black arrows indicate clusters of atoms desorbed by the manipulation.

Figure 6(a) shows the surface observed before the treatment. Figure 6(b) shows a typical time-resolved spectrum, i.e., the tunneling current as a function of time, in which *t*_mes_ = 0 corresponds to the moment when the tunneling current was set to +0.03 nA, similarly to the case shown in figure 3(d). The tunneling current jumped from 0.03 to ∼5 nA and then disappeared. Figure 6(c) shows the STM image after the treatment with the injection time of *t*_i_ = 0.1 s, in which an atomic-scale defect was generated. Namely, the jump in tunneling current shown in figure 6(b) is considered to correspond to the desorption of the surface Zn atom. To confirm this result, we carried out the same process with increased electron injection times *t*_i_ to 0.3 s and 0.5 s, the results of which are shown in figures 6(d) and (e), respectively. Larger defects were generated with increasing *t*_i_, and clusters considered to be formed by the removal of atoms were observed, as indicated by arrows. After the desorption of the Zn atom immediately below the STM tip, the adjacent Zn atoms may have been desorbed depending on the injection time. Further analysis is necessary to determine in detail the conditions required for desorption. Although the lengths of the defects were short, their appearances were very similar to the intrinsic line defect on the ZnTe(110) surface shown in figure 1(a) in that they run along the 〈110〉 direction with single-atomic-row-width on the surface.

Here, the atomic manipulations were demonstrated at 77 K with the same parameter as that used in the previous experiment (figure 3) at 8 K. The higher environmental temperature of 77 K is only different experimental parameter from the former experiment. Considering the two results that the ten times larger tunneling current at 8 K and the higher environmental temperature of 77 K could induce the atomic desorption, environmental temperature may work as an additional driving force to overcome the potential hill of the change in state of the surface Zn atom located just below the STM tip [15], and the atomic desorption might have been induced easier at 77 K than at 8 K. Similar temperature dependence is known for the case of Si(001) surface dimer, where dimer flip-flop motion has temperature dependence [16]. The relationship between atomic desorption and STM parameters is yet unclear. It involves several factors, such as local heating by the tunneling current and the relative position between the tip and surface atoms, which affects the electric field distribution. Further study is necessary to understand the mechanism of the atomic oscillation and desorption.

Finally, we focus on the generated defects. An elliptical disk-shaped dark area was observed around the defects, as shown in figure 6, which is similar to that around the atomic-scale defects and surface steps observed on a GaAs surface [17]. Namely, the results suggest the modulation of the electronic structure in the region, such as charge doping to the host semiconductor [18]. Since a dark area is observed at a negative sample bias voltage, a positively charged dopant center is supposed to be formed here. Although the band structure must be examined in detail by taking into account a tip-induced band bending [18, 19], this atomic-scale fabrication may give a possibility for controlling local electronic structures of this surface.

In fact, in (Ga,Mn)As, for example, activation energy of Mn acceptor was controlled by changing the condition of As vacancies around the Mn atom [20]. On the other hand, ZnTe is known to be a host material of (Zn,Cr)Te, one of the most promising DMSs with intrinsic room temperature ferromagnetism, as mentioned earlier. According to the results of previous studies [21, 22], the magnetic behavior of (Zn,Cr)Te and the electronic states of the host material ZnTe have a close relationship; therefore, the manipulation of the surface electronic states is a key technology for realizing future advances in semiconductor spintronics associated with, for example, nanomagnetism. Although there are many issues to overcome, such as removal of clusters of the desorbed atoms as indicated by black arrows in figures 6(c)–(e), for future application with further optimization and improvement of the manipulation techniques, we may be able to fabricate a desired atomic-scale electronic structure, for example, a local potential modulation, on a surface.

## 4. Conclusions

The charge transfer associated with the surface reconstruction of ZnTe(110) was confirmed in real space at the atomic scale. The tunneling current oscillation, which should be concerned with the positional change of a surface Zn atom between the reconstructed and bulk-like displaced structures as a result of charge injection, was directly observed using STM and was found to be in good agreement with the theoretical calculation of the structural stability. Analysis of the conformational change may provide information on the environmental conditions surrounding a Zn atom. In addition, although the conditions required to induce a conformational change must be clarified, atomic-scale defects were successfully generated below the STM tip using the atom manipulation process based on charge injection. Since the generated defects have similar characteristics to such as the charge dopant in a host semiconductor, the technique of precise atom manipulation may be a key factor in the future development of spintronics or nanomagnetics based on DMS materials.
